# Impact of the Preparation Procedure on the Performance of the Microporous HKUST-1 Metal-Organic Framework in the Liquid-Phase Separation of Aromatic Compounds

**DOI:** 10.3390/molecules25112648

**Published:** 2020-06-06

**Authors:** Vera I. Isaeva, Bulat R. Saifutdinov, Vladimir V. Chernyshev, Vadim V. Vergun, Gennady I. Kapustin, Yulia P. Kurnysheva, Mikhail M. Ilyin, Leonid M. Kustov

**Affiliations:** 1 N.D. Zelinsky Institute of Organic Chemistry, Russian Academy of Sciences, Leninsky Prospect 47, 119991 Moscow, Russia; polubrat@mail.ru (V.V.V.); gik@ioc.ac.ru (G.I.K.); lmkustov@mail.ru (L.M.K.); 2 Institute of Ecotechnology, National University of Science and Technology MISiS, Leninsky Prospect 4, 119991 Moscow, Russia; 3 A.N. Frumkin Institute of Physical Chemistry and Electrochemistry, Russian Academy of Sciences, Building 4, 31 Leninsky Prospect, 119071 Moscow, Russia; vladimir@struct.chem.msu.ru (V.V.C.); kurnyshevay@mail.ru (Y.P.K.); 4 Department of Chemistry, M.V. Lomonosov Moscow State University, 1-3 Leninskie Gory, 119991 Moscow, Russia; 5 A.N. Nesmeyanov Institute of Organoelement Compounds, Russian Academy of Sciences, 28 Vavilov St., 119991 Moscow, Russia; mil@ineos.ac.ru

**Keywords:** metal-organic frameworks (MOFs), selective adsorption, high-performance liquid chromatography

## Abstract

To date, metal-organic frameworks (MOFs) have been recognized as promising solid phases in high-performance liquid chromatography (HPLC). This research aimed to elucidate the role of the physico-chemical characteristics of the microporous HKUST-1 metal-organic framework in its operation as a selective adsorbent in HPLC. For this, the HKUST-1 samples were prepared by microwave-assisted synthesis and a solvothermal procedure. According to the chromatographic examinations, the HKUST-1 material synthesized in the microwave fields shows an efficient performance in the selective adsorption of aromatic compounds with different functionalities. This study revealed a significant impact of the preparation procedure on the mechanism of the liquid-phase adsorption on the HKUST adsorbents under conditions of the HPLC. An effect of the elution solvent with the different coordination ability to the Cu^2+^ sites in the HKUST-1 structure on the adsorption selectivity was observed.

## 1. Introduction

Currently, high-performance liquid chromatography (HPLC) is a powerful tool for the selective and preparative separation of a large variety of organic compounds [1]. Packing materials are of a primary importance for the efficient operation of chromatographic columns [2], therefore, the need for advanced adsorbents as stationary phases in HPLC method is rapidly increasing. Besides the usage of these adsorbents for liquid-phase separations under analytical and preparative HPLC conditions, these adsorbents can be used in static liquid-phase adsorption techniques such as solid-phase extraction, selective adsorption, deep purification of substances, etc.

To date, a number of different porous supports have been used for HPLC separation. The essential characteristics of a HPLC support can be divided into two main groups: geometry and surface chemistry. The geometry of the column material comprises the following characteristics: surface area, pore volume, pore diameter, particle size and shape. Among them, the surface area of HPLC adsorbents is regarded as one of the most important parameters, because the retention volume is generally proportional to the surface area accessible for the molecules of a given analyte [3]. Surface area accessibility is dependent on the analyte molecular size, adsorbent pore diameter, and pore size distribution. Other important factors are the support high porosity, support morphology and crystal size associated with homogeneous size distribution [3]. In particular, the particle size, particle shape, particle size distribution, packing density, and packing uniformity impact on the column efficiency and flow resistance. In its turn, the surface functionality (along with specific surface area) affects the analyte retention and separation selectivity. Moreover, the specific surface area impacts on the adsorbent capacity under conditions other selective liquid-phase adsorption techniques, such as solid-phase extraction and purification of substances. The surface functionality of the support material defines the main type of chemical interactions of the surface with the eluent and analyte molecules. Note an interrelation of different characteristics of the column materials. The variations of the porosity including the pore diameter can affect both the adsorbent surface area and the bonding density. The type of the support material affects adsorbent surface chemistry [3].

In this context, a novel type of the hybrid nanoporous materials, namely metal-organic frameworks or MOFs, is regarded as promising stationary phases for HPLC having both favorable geometrical parameters and rich surface chemistry. MOF matrices (porous coordination polymers by nature) with two- or three-dimensional highly ordered structures are composed of metal ions or metal-oxo units (secondary building units [SBUs]) and organic polytopic molecules (linkers) in a long-range order, resulting in permanent, porous, open crystalline frameworks [4,5,6,7]. MOF materials are synthesized by self-assembly of organic and inorganic building blocks according to the principles of modular chemistry [8]. Therefore, metal-organic frameworks with high specific surface areas and porosities designated for the specific potential applications including HPLC can be constructed through the selection of different SBUs and organic linkers. The metal ions in the MOF matrices influence their adsorption properties and therefore the separation selectivity [9]. For instance, partial positive charges on the metal sites in metal-organic frameworks may improve the adsorption selectivity for the separation of compounds with different polarities [10,11].

The applicability of MOF matrices as packing materials for HPLC columns may arise from easy and uniform diffusion pathways associated with the ordered pore structure including controllable and predictable pore size and geometries [12,13]. In recent years, several MOF materials were explored as stationary phases in HPLC and demonstrated their efficiency [11,14]. In HPLC, the separation mechanism of the MOF matrices is based on the molecular sieving effect, van der Waals interactions, and specific groups interactions [7,15]. In particular, matching of the size and shape of the analytes and the MOF pores also contribute to the separation [16].

Another important property, which is lacking in inorganic stationary phases, is a metal-organic framework flexibility [17]. Thus, besides geometry and surface chemistry, the selectivity of chromatographic separation on MOF materials is defined by the structural characteristics, including network flexibility or breathing effects [18,19]. Previously, we have reported the utilization of the flexible microporous MIL-53(Al) framework with breathing properties as a stationary phase for HPLC for selective adsorption of mono-, bi-, and tricyclic aromatic molecules [20]. The MIL-53(Al) and MIL-47(V) adsorbents were investigated for the separation of aromatic isomers using hexane and dichloromethane–hexane binary systems as the mobile phases. The separation performance of these metal-organic stationary phases depended on the combined mechanisms based on π-π stacking interactions, hydrogen bonding, size-exclusion, shape selectivity, coordination interactions, and hydrophobicity [15,21,22].

To date, one more type of MOF adsorbent, i.e., microporous HKUST-1 metal-organic framework (Cu_3_(BTC)_2_, BTC = benzene-1,3,5-tricarboxylate) [23,24] has been recognized as a rather promising stationary phase for HPLC based on its interaction with π-electron systems [25,26]. Additionally, a number of the characteristics of the HKUST-1 material help in this application, such as a large surface area and high porosity and open Cu^2+^ metal sites, which are Lewis sites providing selective adsorption, in a combination with weak Brønsted sites in organic linkers [27,28,29].

Tuning the HKUST-1 porous structure for an enhancement of its performance in HPLC was demonstrated in [30]. Macroporous HKUST-1 microparticles prepared by a solvothermal etching strategy with hydroquinone were studied in the separation of styrene and ethylbenzene. The same researchers prepared a HKUST-1 monolith with a hierarchical porous structure including interconnected macropores [31]. Due to their favorable texture characteristics, the HKUST-1 monoliths demonstrated fast HPLC separation.

In this work, we intend to elucidate an impact of the intrinsic properties of the HKUST-1 adsorbent such as the porous structure and functionality (particularly, the presence of open metal sites) on its operation in HPLC. For this purpose, some functional characteristics of the HKUST-1 material were compared with those of the MIL-53(Al) structure previously studied in HPLC [20,21]. The main difference between HKUST-1 and MIL-53(Al) metal-organic frameworks is the presence of the OH vertices in the MIL-53(Al) structure, which results in a highly flexible character of this network and therefore, a pronounced “breathing” effect manifested as a pore diameter change [32,33,34]. As to the pore system, the MIL-53(Al) porous structure is arranged as a fully open system of one-dimensional channels, while the HKUST-1 framework contains a three-dimensional channel system. The channels in the HKUST-1 matrix consist of two sorts of the cavities of ~1.4 nm pore diameter and ~1.1 nm pore diameter connected through pores of ~1.0 nm diameter [35]. There are also smaller ~0.5 nm diameter cavities in the HKUST-1 structure located between the larger cavities [36].

It has been established that the preparation method can impact significantly on a number of essential characteristics of MOF materials, such as morphology, particle size and shape, textural properties, etc. Through a careful choice of synthesis conditions, it is possible to control the structure, texture, and morphology of the resulting framework and therefore its functional properties for the specific applications, including liquid-phase separations under conditions of HPLC, solid-phase extraction, purification of substances, etc. [37,38].

To date, a number of methods for the rapid preparation of the HKUST-1 material were elaborated, which include microwave (MW) heating, ultrasound treatment, mechanochemistry, and solvothermal synthesis [39,40,41,42,43,44,45,46,47]. However, significant research efforts are still directed towards the optimization of the structural and textural characteristics of the HKUST-1 product through an appropriate synthetic strategy [46,48,49,50].

Among these methods, a microwave-assisted technique has been introduced as a more rapid way to reduce the synthesis time with increasing the production rate [51]. It has been established that the MW-synthesis can provide the fast kinetics of crystal nucleation, crystal growth, and high yields in the production of MOF materials [52]. This technique allows one to control the particle size distribution, phase selectivity, and macroscopic morphology in the synthesis of nanoporous materials [53,54,55]. As to the HKUST-1 structure, the comparative study of the MW-procedure versus a conventional solvothermal method was reported that shows the advantages of the MW-assisted synthesis, i.e., a much shorter time and lower reaction temperature [47].

Keeping this picture in mind, we decided to verify the impact of the preparation procedure on the adsorption selectivity of the HKUST-1 metal-organic framework as the packing material for the HPLC columns, cartridges for the liquid-phase separations, and other techniques of liquid-phase adsorption, such as solid-phase extraction and purification. For this purpose, we have synthesized of a number of HKUST-1 samples with appropriate particle sizes using MW-activation of the reaction mass and a solvothermal procedure by changing the synthesis parameters.

In order to study the adsorption selectivity of the HKUST-1 samples under liquid-phase conditions, we selected the HPLC technique. The selectivity of the HKUST-1 material synthesized in MW-fields was studied using a representative number of the individual organic compounds including physiologically active ones (Table 1) and elution solvents with the different coordination abilities for the Cu^2+^ sites in the metal-organic structure. The comparative study of both samples obtained in MW-fields and under solvothermal conditions was carried out in the benzene adsorption from its solutions in the *n*-hexane—isopropanol binary system. The dependencies of the volumetric flow rates of the elution solvent as well as column temperature on the benzene dynamic adsorption on the HKUST-1 samples in HPLC were also examined.

The synthesized HKUST-1 samples were characterized by PXRD, SEM, and N_2_ low temperature adsorption.

## 2. Results

### 2.1. HKUST-1 Material Synthesis

It is known that MW-radiation affects both the rapid crystal nucleation and the crystal growth [56,57], so our research focus was placed on providing the MW-conditions, under which the crystal growth would dominate, because rather large crystals with micrometric sizes and homogeneous distribution on sizes should be used for HPLC column packing. In particular, the importance of the appropriate solvent for the growth of MOF crystals with the targeted structure, morphology, and dispersion is well documented [58]. The HKUST-1*mw* sample was synthesized using a DMF–H_2_O system.

Different solvent systems were tested in the solvothermal procedures for the preparation of the HKUST-1 materials. The HKUST-1*solv.1* sample was prepared in the mixed solvent EtOH–H_2_O. In order to prepare the HKUST-1 solvothermal material under the same conditions as the HKUST-1*mw* sample (except the heating mode), HKUST-1*solv.2* was prepared by the original procedure involving the synthesis time shortening till 30 min and using DMF–H_2_O mixture.

### 2.2. Characterization of HKUST-1 Materials

**Figure 1:**
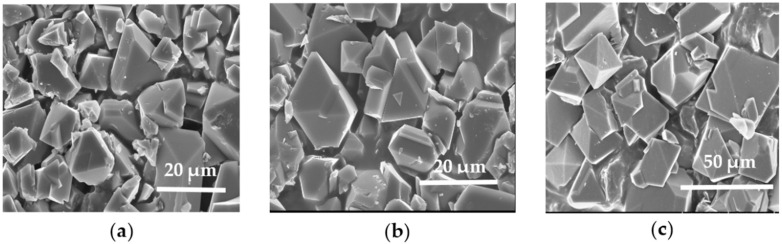
SEM micrographs of the HKUST-1*solv.1* (**a**), HKUST-1*solv.2* (**b**) and HKUST-1*mw* (**c**) samples.

The HKUST-1 materials synthesized under solvothermal conditions are composed of tetrahedral crystals with average diameters of approximately 5–15 μm for the HKUST-1*solv.1* sample (Figure 1a) synthesized in the ethanol-H_2_O system. The HKUST-1*solv.2* sample prepared in the DMF-H_2_O mixed solvent has crystals with the sizes in the range ~5–20 μm, so the solvent system does not impact remarkably on size and morphology of the HKUST-1 material synthesized under solvothermal conditions.

Using MW-radiation for the HKUST-1 synthesis results in the formation of larger crystals with the almost ideal tetrahedral shape and sizes of around 25–40 μm (Figure 1b). Note that the HKUST-1*mw* sample features a more homogeneous distribution of crystals in size and shape. Probably, the crystal growth rate prevails over the rate of the nucleation under conditions of MW-radiation. Note, the similar crystal sizes of HKUST-1 samples obtained by MW-assisted solvothermal synthesis were observed previously [47].

### 2.3. Structural Examinations

**Figure 2:**
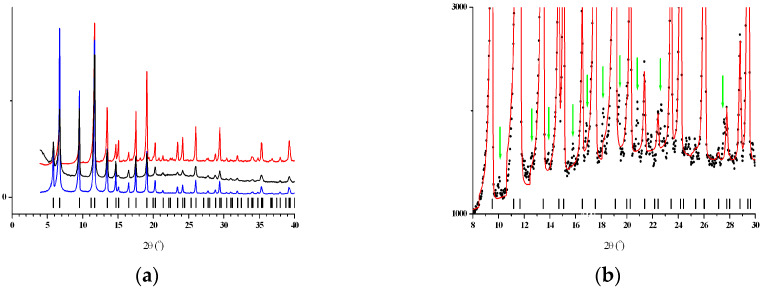
(**a**) The combined presentation of the XRD patterns of the HKUST-1*mw* (red), HKUST-1*solv.1* (blue), and HKUST-1*solv.2* (black) samples. The vertical black bars denote the positions of the peaks calculated for the cubic unit cell in the space group Fm-3m. (**b**) The experimental (black dots) and calculated (red line) powder XRD patterns of the HKUST-1*mw* sample. The vertical black bars denote the positions of the peaks calculated for the cubic unit cell in the space group Fm-3m. The vertical green arrows show the positions of some additional weak peaks.

Powder patterns of three samples—HKUST-1*mw*, HKUST-1*solv.1*, and HKUST-1*solv.2*—are typical for the cubic unit cell with *a* = 26.277, 26.292, and 26.305 Å, respectively, in the space group Fm-3m. However, after the careful inspection of the HKUST-1*mw* powder pattern, we discovered weak additional peaks, as shown in Figure 2b, which can be indexed in the same cubic cell though in the Pm-3m space group. Thus, we can state that microwave irradiation led to symmetry distortions in the HKUST-1 sample.

Note that the HKUST-1*solv.2* material was synthesized under the same synthesis parameters and reaction mixture composition as those used for the HKUST-1*mw* sample, except for the heating mode. Thus, the new phase formation can only be assigned to the MW-radiation effect, so this synthesis route may result in a distinct phase, which has not been observed in conventional solvothermal syntheses using an identical reaction mixture.

### 2.4. Textural Properties of HKUST-1 Materials

**Figure 3:**
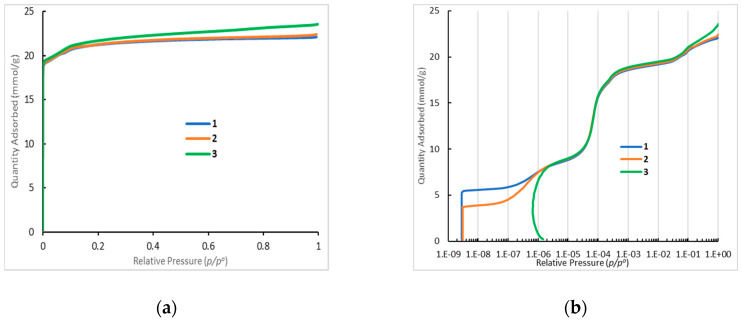
Complete N_2_ adsorption isotherms (**a**) and their initial sections (**b**) for the HKUST-1*mw* (1), HKUST-1*mw* pressurized at 20 atm (2), and HKUST-1*solv.1* (3) samples measured at 77 K.

**Figure 4:**
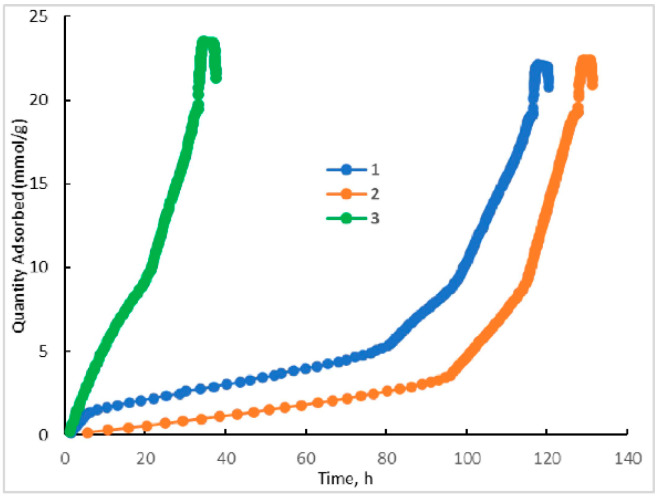
The time of the achievement of the adsorption equilibrium on the different isotherm plots for the HKUST-1*mw* (1), HKUST-1*mw* pressurized material (2), and HKUST-1*solv.1* (3).

**Figure 5:**
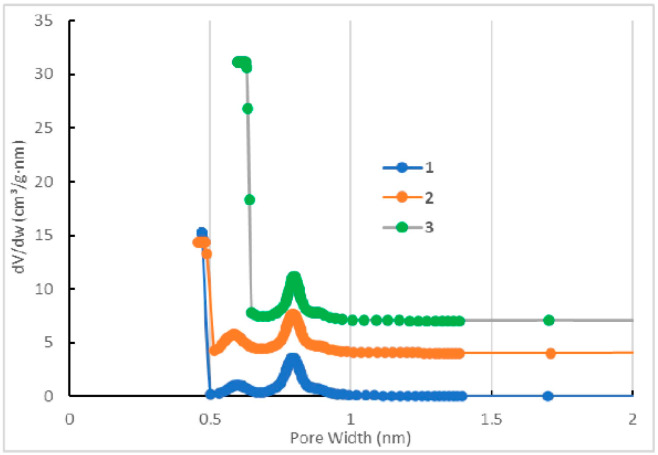
The distribution of the micropore volume on the sizes calculated by the Horvath–Kawazoe method for the HKUST-1*mw* (1), HKUST-1*mw* pressurized (2), and HKUST-1*solv.1* (3) samples.

N_2_ low-temperature adsorption reveals that the preparation procedures affect to some extent the textural characteristics of the HKUST-1*mw* and HKUST-1*solv.1* samples (Table 2). However, the HKUST-1*solv.1* material has a larger pore width and a bit larger fraction of mesopores than the HKUST-1*mw* matrix. The adsorption curves for the HKUST-1*mw*, HKUST-1*solv.1*, and HKUST-1*solv.2* materials (Figure 3) correspond to the Type I characteristic to the microporous matrices. The stepped adsorption isotherm measured for the HKUST-1 samples is observed at low pressures and corresponds to micropore filling (Figure 4). Note that the N_2_ adsorption rate on the HKUST-1 materials is almost negligible at very low pressures. This fact could be explained by the presence of two types of micropores in the HKUST-1 material (Figure 5). The curve of the pore size distribution indicates that the narrow micropore diameter is ~0.4–0.6 nm. The sizes of the “windows” in the primary structural elements fit to the size of the adsorbed N_2_ molecule (~0.2 nm). Their filling proceeded for more than 100 h. On the contrary, the wide micropores (~0.8 nm) are filled faster. The stepped isotherm shape for HKUST-1 materials at low relative pressures is in a good agreement with the experimental results obtained in the work of Schlichte [40].

From adsorption data, the difference between the HKUST-1*solv.1* and HKUST-1*mw* samples could be explained by the assumption that very narrow micropores are not formed in the HKUST-1*solv.1* material in contrast to the HKUST-1*mw* matrix. Possibly, this difference can affect to some extent the adsorption characteristics of both adsorbents in HPLC.

In order to evaluate the mechanical robustness of the material HKUST-1*mw* and the possibility of its shaping and compacting needed for some practical applications, its textural properties upon pressing were probed. The HKUST-1*mw* sample was pressurized under 20 atm, further its N_2_ low-temperature adsorption was measured. This test showed that the N_2_ adsorption isotherms for the HKUST-1*mw* sample without pressing and after pressing are almost identical and there are almost no differences in the textural characteristics (Table 2).

The differences for these two samples are observed under very low pressures only (Figure 3 and Figure 4). They are expressed in the different times of the achievement of the adsorption equilibrium at filling of the primary structure elements (*p*/*p*_0_ = 10^−9^–10^−7^). The rate of the achievement of the adsorption equilibrium for the sample pressurized under 20 atm is 1.5 times higher than for the parent HKUST-1*mw* material. The results on probing the mechanical stability of the HKUST-1 material can expand its potential application fields including adsorption.

### 2.5. Selectivity of Aromatic Molecules Liquid-Phase Adsorption onto the HKUST-1mw Material upon HPLC

Among other important factors, such as π-π-stacking interactions, the selective adsorption on HKUST-1 metal-organic framework is determined by a possibility of a desorption of the solvent pre-adsorbed on Cu^2+^ ions and subsequent interactions of the adsorbate molecules with these active sites. Note, a possibility of the pore expansion in the HKUST-1 structure due to adsorption (analogously to the breathing of the MIL-53(Al) matrix) does not exist. On the other hand, contrary to the MIL-53(Al) structure, which has no coordinatively-unsaturated sites, such as metal ions, the HKUST-1 framework has Cu^2+^ ions as inorganic nodes with some adsorption activity. Therefore, HKUST-1 is mainly a hydrophilic adsorbent and MIL-53(Al) is a hydrophobic adsorbent [24]. As a result, molecules of polar solvents, such as methanol, can be adsorbed, and its adsorption displacement with molecules of other type adsorbates is difficult.

As it can be seen from Table 1, the studied compounds are weakly adsorbed on the HKUST-1*mw* stationary phase from methanol. This fact can be explained taking into account blocking of the adsorption sites in the HKUST-1 framework with pre-adsorbed methanol. Therefore, 4-chlorophenol (Compound 1) is adsorbed from acetonitrile much stronger than from methanol, because acetonitrile, probably, does not block the HKUST-1 active sites. Moreover, phenols, obviously, are solvated by methanol more strongly than by acetonitrile due to the presence of hydroxyl groups in phenol and methanol molecules, so stronger solvation of phenols in methanol medium decreases the retention on the HKUST-1 material under HPLC conditions. Substituted phenols, chloro- and dichlorophenols containing one or two methyl groups are adsorbed weaker from methanol and acetonitrile than 4-chlorophenol. This fact could be explained by the steric hindrances for the transport of organic molecules in the framework narrow pores (mesopores with small sizes and even large micropores). The HKUST-1 matrix does not feature “breathing” unlike the MIL-53(Al) structure. Therefore, this network is not capable of deformations due to adsorption of specific molecules. This adsorptive deformation involves tuning the pore geometry for the shape and size of the adsorbate molecules. Therefore, steric obstacles are a significant factor excluding the penetration of bulky adsorbate molecules in the HKUST-1 micropores, despite a larger pore size, than in the case of the MIL-53(Al) framework [20,59].

Simultaneously, the HKUST-1*mw* adsorbent features a sufficiently high structure selectivity for the separation of dichloro-, trichloro-, and nitrophenols by eluting with acetonitrile (Table 1). Tetracycline is adsorbed weakly on the HKUST-1*mw* material due to inaccessibility of the framework narrow pores for these molecules. A weak retention of tetracycline is caused, probably, by adsorption in the mesopores of the framework or on the outer surface of the crystallites. Note that a small fraction of mesopores is present in the porous system of both samples despite a microporous character of the HKUST-1 matrix (Table 2).

Tropic acid and ibuprofen are adsorbed weakly also on the HKUST-1*mw* material. In particular, ibuprofen is retained weaker than tropic acid that is structurally close to ibuprofen molecules due to steric hindrances for the penetration in the narrow pores of this metal-organic matrix.

Along steric factors, the presence of acid and basic centers in the adsorbate molecules plays an important role in liquid-phase adsorption on the HKUST-1 material. For instance, paracetamol and diphenylamine containing an N atom are adsorbed rather strongly on this metal-organic matrix. An interaction of the lone electron pair of nitrogen with carboxyl groups in the inorganic linkers or Cu^2+^ ions of the metal-organic framework results in a stronger adsorption from these adsorbate solutions. Additionally, paracetamol and diphenylamine molecules feature a more planar geometry than tropic acid and ibuprofen molecules. Therefore, the penetration of paracetamol and diphenylamine molecules and their binding with adsorption sites in the HKUST-1 pores are more preferential.

Most of the compounds studied are more strongly adsorbed on the HKUST-1 matrix from the acetonitrile medium. On the contrary, nicotine and benzotriazole are adsorbed stronger from a methanol solution. In this case, probably, the displacement of pre-adsorbed methanol molecules by these organic compounds from the adsorption sites (Cu^2+^ ions) in the HKUST-1 framework occurs due to the presence of the pyridine-type N atom in their molecules, so pyridine-type N atom contains a lone electron pair that is basic and can interact with HKUST-1 open metal sites (Cu^2+^) due to their acidic properties [27,28]. Lone electron pair of N atom in other molecules such as paracetamol or diphenylamine is involved in conjugation, therefore basic properties of these compounds are weaker and insufficient for the displacement of pre-adsorbed methanol molecules. Simultaneously, strong solvation of nicotine and benzotriazole in the acetonitrile medium hinders the liquid-phase adsorption of these molecules on the HKUST-1*mw* adsorbent by elution with acetonitrile.

### 2.6. Study of the Mechanism of Liquid-Phase Adsorption onto HKUST-1 Materials

The nature and composition of the elution solvent are crucial factors that determine the selectivity of the liquid-phase adsorption onto the MOF adsorbents (and not only MOF stationary phases) under HPLC conditions. All studied aromatic organic compounds are weakly retained from polar solvents on the HKUST-1 adsorbent. This fact could be explained by a pronounced adsorption of these solvents themselves on the open metal sites (Cu^2+^) ions of the HKUST-1 metal-organic framework, so a competitive adsorption of the molecules of the studied aromatic compounds and solvents occurs on the adsorption sites of this matrix. Therefore, we studied an HPLC elution using HKUST-1 samples and the solvent system composed of the non-polar component, e.g., *n*-hexane as a main fraction, and a polar component, i.e., isopropanol as a minor fraction.

In order to elucidate the liquid-phase adsorption mechanism for the HKUST-1*mw* and HKUST-1*solv.1* samples under HPLC conditions, we have studied the dependencies of the benzene retention on the mobile phase composition, mobile phase flow rate, and column temperature using *n*-hexane–isopropanol mixed eluents. We have taken into account the dependencies of the retention factor on the composition and flow rate of the mobile phase as well as a distribution constant (in logarithmic units) on the temperature.

To calculate the retention factor, we used the values of the dead volume (*V*_M_) of the column obtained according to the minor disturbance method [60] with a mobile phase flow rate *F* = 0.05 mL/min. They are *V*_M_ = 0.524 mL for the column packed with HKUST-1*solv.1* and *V*_M_ = 0.369 mL for the column with HKUST-1*mw*. We used these dead volume values for the studied columns, because they are significantly lower than dead volume values obtained by the weight method [60]. Note that in the latter case, these values are *V*_M_ = 0.627 mL for the column packed with HKUST-1*solv.1* and *V*_M_ = 0.664 mL for the column with HKUST-1*mw*. This fact could be explained by an inaccessibility of some fraction of the adsorbent voids in the framework structure for the molecules from the mobile phase even at a flow rate as high as 0.05 mL/min. Probably, these voids are pores with the sizes too narrow for the penetration of *n*-hexane and isopropanol molecules.

**Figure 6:**
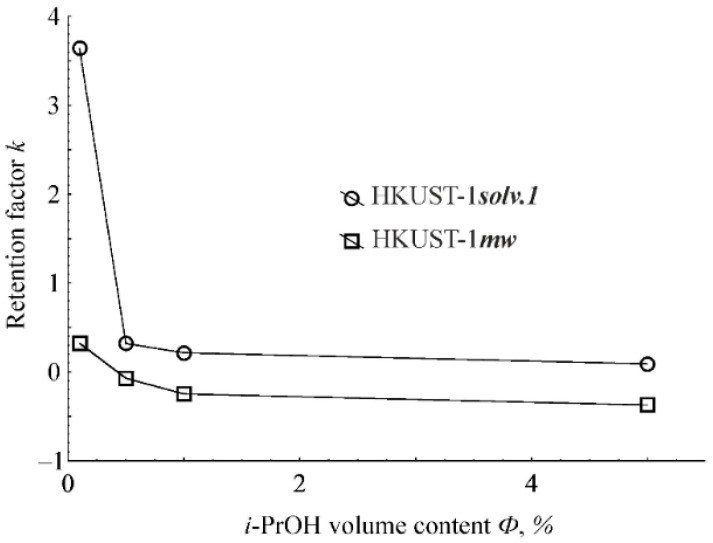
Dependences of the benzene retention factor on the *i*-PrOH content (vol %) in the mobile phase for the HKUST-1*mw* and HKUST-1*solv.1* samples.

Figure 6 shows the dependencies of the retention factor on the volume content of isopropanol in the mobile phase for the benzene adsorption on the HKUST-1*mw* and HKUST-1*solv.1* samples. With increasing isopropanol content in the mobile phase, the benzene retention decreases significantly due to the solvation of benzene molecules with isopropanol molecules as well as blocking of the free adsorption sites of the HKUST-1 structure with isopropanol molecules. At high contents of *n*-hexane in the mobile phase, benzene retains extremely strong, because adsorption sites are occupied by *iso*-propanol molecules to a lesser degree. Therefore, benzene molecules can interact not only with π-systems of the organic linkers but with open metal sites of the HKUST-1 structure. Obviously, an increase of the *iso*-propanol content in the mobile phase results in a blockage of the open metal sites due to adsorption of alcohol molecules.

With an increase of the *n*-hexane content, the benzene retention increases much stronger on the HKUST-1*solv.1* material than on the HKUST-1*mw* adsorbent. Moreover, generally, the benzene adsorption on the HKUST-1*solv.1* sample is stronger than on the HKUST-1*mw* material. In case of benzene adsorption on the HKUST-1*mw* sample, even negative values of the retention factor are observed with an increase of the isopropanol content in the mobile phase. This phenomenon indicates a negative benzene adsorption, in other words, *n*-hexane and isopropanol are adsorbed on the HKUST-1*mw* material stronger than benzene itself. It is interesting to note that benzene is adsorbed on the HKUST-1*solv.1* sample many times stronger than on the HKUST-1*mw* material at the maximal content of *n*-hexane in the mobile phase. We suggest that this phenomenon could not be explained by a smaller particle size and therefore by a higher dispersion of the HKUST-1*solv.1* sample, because the adsorption on the external surface of the granules impacts insignificantly on the retention. Therefore, we decided to elucidate a possibility of the penetration of benzene molecules in the narrow pores, i.e., mesopores with small sizes (around 2 nm) and micropores with wide widths (0.8–0.9 nm) in the HKUST-1 structure (Table 2). For this purpose, the influence of the volumetric flow rate of the mobile phase on the benzene retention factor during its adsorption onto the HKUST-1*solv.1* and HKUST-1*mw* samples was studied.

**Figure 7:**
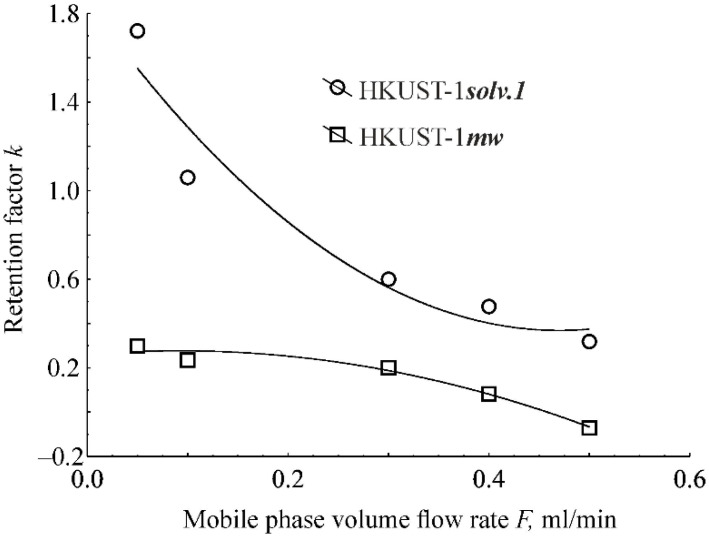
Dependences of the benzene retention factor on the volumetric flow rate of the mobile phase for the HKUST-1*mw* and HKUST-1*solv.1* samples.

The dependencies of the benzene retention factors on the volumetric flow rate of the mobile phase for the HKUST-1*solv.1* and HKUST-1*mw* samples are shown in Figure 7. In the case of the column packed with the HKUST-1*solv.1* sample, the retention increases with a decrease of the mobile phase flow rate. The curve of this dependence has a concave shape without reaching a plateau even at a flow rate of 0.05 mL/min. When the column is packed with the HKUST-1*mw* adsorbent, the dependence of the retention factor on the mobile phase flow rate has a convex shape and reaches a plateau at the flow rates 0.05–0.1 mL/min. The fact that the benzene retention factor for the column packed with the HKUST-1*mw* sample becomes a positive value with a decrease of the mobile phase flow rate indicates the possibility of the penetration of benzene molecules at low flow rates into narrow pores that are inaccessible for the adsorbate at higher flow rates. In case of a high rate of the mobile phase flow through a microporous adsorbent layer in the column, benzene molecules could not penetrate in the narrow HKUST-1*mw* pores due to a delayed adsorbate mass transport, which results in the accelerated adsorbate elution from the column. The retention factor would not depend on the flow rate under condition of the equal access of the adsorbate molecules in the HKUST-1*mw* narrow pores at all volumetric flow rate values. Probably, an easy penetration of benzene molecules in the HKUST-1*mw* pores and adsorption in these pores is achieved only at low flow rates, i.e., 0.05–0.1 mL/min.

In case of the HKUST-1*solv.1* adsorbent, the curve of the dependence of the benzene chromatographic retention factor on the volumetric flow rate of the mobile phase does not reach a plateau even in the range of flow rates around 0.05–0.1 mL/min (Figure 7). Consequently, the novel adsorption sites continue to open for benzene molecules in the HKUST-1*solv.1* sample with a decrease of the mobile phase flow rate. Such a difference may be related with the differences in the textural properties of both supports (Table 2). However, we cannot suggest what type of HKUST-1 pores becomes accessible for benzene molecules at low flow rates of the mobile phase, i.e., micropores with wide widths (0.8–0.9 nm) or a small fraction of the mesopores.

The mechanism of the liquid-phase benzene adsorption was studied under conditions of extremely low degrees of the coverage of the adsorbent surface (the so-called Henry range), because most HPLC techniques are realized upon adsorption in the Henry range. Moreover, the differences for studied two HKUST-1 samples are namely appeared in the Henry range, because during N_2_ low temperature adsorption they are observed under very low pressures only (Figure 3 and Figure 4). Adsorbate molecules are adsorbed preferentially in the stationary phase micropores featuring a highest energy during adsorption on the microporous solids in the Henry range. If the space of a micropore is inaccessible for the adsorbate molecules due to steric hindrances, these molecules can be adsorbed on the surface of mesopores with very small sizes [61].

**Figure 8:**
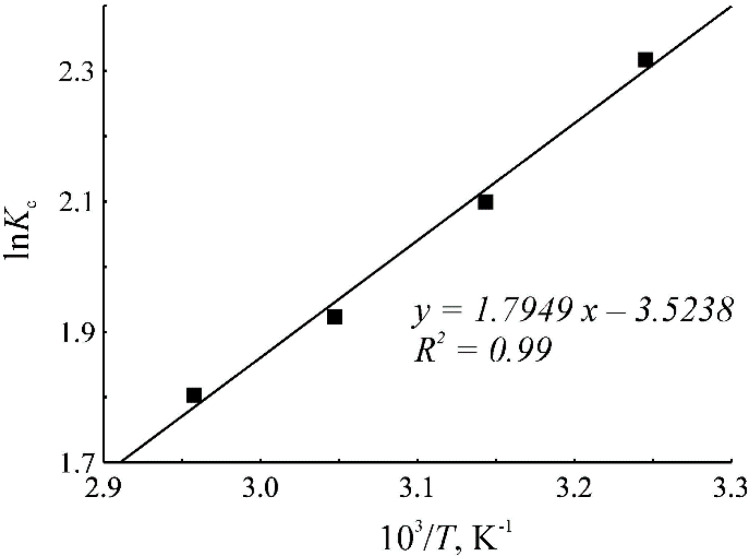
Dependence of the logarithm of the distribution constant of benzene on the inverse temperature during adsorption onto the HKUST-1*mw* sample.

For a more detailed study of the mechanism of the liquid-phase adsorption, it would be necessary to measure the thermodynamic characteristics of benzene adsorption on the HKUST-1*solv.1* and HKUST-1*mw* materials. From the technical point of view, it is almost impossible to perform the HPLC tests with flow rate values lower than 0.05 mL/min. Assuming that the system achieves an adsorption equilibrium at this flow rate value, the thermodynamics of the benzene adsorption was studied on the HKUST-1*solv.1* and HKUST-1*mw* samples. In order to calculate the enthalpy and entropy of adsorption, we used processing of the dependence of the logarithm of the distribution constant ln*K*_c_ on the inverse temperature according to a regression analysis. The distribution constant was calculated according to the previous works [62,63,64,65]. The dependence of the logarithm of the distribution constant on the inverse temperature during benzene adsorption from pure *n*-hexane on the HKUST-1*mw* sample is given in Figure 8. The adsorption is weakening with an increase of the column temperature. The enthalpy (∆*H*°) of benzene adsorption from *n*-hexane on the HKUST-1*mw* sample is −14.92 kJ/mol, and the adsorption entropy (∆*S*°) is −29.28 J/(mol K). Thus, the liquid-phase adsorption on the HKUST-1*mw* sample has an exothermic character with a pronounced localization of the molecules in the framework on the surface of pores. This pattern is similar to the liquid-phase adsorption on the surface of traditional mesoporous stationary phases for HPLC, such as silica gels, polymeric, and carbon materials [63,64,65].

On the contrary, the chromatographic retention during benzene adsorption from pure *n*-hexane on the HKUST-1*solv.1* sample increases with a temperature increase. So, in this case, the adsorption is an endothermic process. The enthalpy of benzene adsorption from *n*-hexane on the HKUST-1*solv.1* sample is ∆*H*° = +28.35 kJ/mol, the adsorption entropy is ∆*S*° = +102.92 J/(mol K). Probably, the mechanism of the benzene liquid-phase adsorption on the HKUST-1*solv.1* sample is similar to the entropy-driven mechanism of the liquid-phase adsorption of aromatics into the micropores of the MIL-53(Al) metal-organic framework [59]. Obviously, it means that benzene molecules with a critical diameter of 0.66 nm can penetrate into micropores of the HKUST-1*solv.1* material. Probably, in case of the adsorption on the HKUST-1*mw* matrix, benzene molecules cannot penetrate into its micropores due to their smaller size (0.4–0.8 nm) as compared to the solvothermal sample (0.6–0.9 nm, Table 2). So, in case of the HKUST-1*solv.1* material, benzene molecules are adsorbed mainly on the surface of very small mesopores with sizes around 2 nm.

The dependencies of the logarithm of the distribution constant ln*K*_c_ on the inverse temperature has an extreme character during benzene adsorption onto both HKUST-1 samples from a *n*-hexane–*iso*-propanol binary mixture with the *n*-hexane to *iso*-propanol ratio of 99.5/0.5%. This pattern reflects the change of the mechanism of the adsorption on the HKUST-1*solv.1* and HKUST-1*mw* samples through a variation of the temperature of the adsorption system. Therefore, the experimental data are not linearized in the coordinates ln*K*_c_–1/*T*, which makes impossible to calculate the adsorption enthalpy and entropy values upon adsorption from *n*-hexane–*iso*-propanol medium.

## 3. Discussion

Our study has revealed the pronounced impact of the preparation procedure of the HKUST-1 material on the mechanism of the liquid-phase adsorption of aromatic molecules onto its surface. Probably, the differences in the adsorption mechanisms for the samples prepared by the different methods could be associated with more favorable diffusion pathways in the HKUST-1*solv.1* matrix due to its smaller crystal size, which reduces diffusion limitations, as well as due to a larger micropore size and a bit larger mesopore fraction.

A significant impact of the preparation procedure on the performance of the HKUST-1 materials in the HPLC was observed. The solvothermal HKUST-1 sample with smaller crystals and a larger micropore width shows more efficient benzene adsorption than its MW-counterpart. Possibly, the dispersion and porous structure (rather a hierarchical one) of the solvothermal material contribute to the optimal diffusion pathways and mass transfer of the adsorbed benzene molecules. A comprehensive study of the mechanism of the liquid-phase adsorption shows the possibility of the penetration of benzene molecules into the micropore space in the case of the HKUST-1*solv.1* material synthesized by the solvothermal technique, and respectively localization of benzene molecules on the surface of pores of the HKUST-1*mw* sample. The liquid-phase benzene adsorption by the HKUST-1 material synthesized by the solvothermal technique features an entropy-driven mechanism because of the endothermic pattern of the adsorption process. On the contrary, the liquid-phase adsorption on the HKUST-1*mw* matrix is characterized by the “classical” enthalpy-driven mechanism.

The HKUST-1 material synthesized by the solvothermal procedure due to accessibility of its micropores for analyte molecules can be used for static liquid-phase adsorption techniques, such as selective adsorption, solid-phase extraction, purification of substances. On the other hand, the HKUST-1 matrix prepared by the MW-assisted synthesis can be used for the liquid-phase separation by HPLC due to the predominant adsorption of the analyte on the surface of its pores.

## 4. Materials and Methods

All reagents and solvents employed were commercial products (Acros Organics, Antwerpen, Belgium). *N,N*’-dimethylformamide (DMF) was distilled over CaH_2_ under a reduced pressure.

### 4.1. Preparation of HKUST-1 Materials

#### 4.1.1. HKUST-1*mw* Sample

Cu(NO_3_)_2_ 3H_2_O (8.58 mmol) was dissolved in H_2_O, while H_2_BTC (4.76 mmol) was dissolved in absolute DMF (20 mL). Both solutions were stirred for 20 min on a magnetic stirrer, then the mixture was transferred into a glass ampoule and heated at an atmospheric pressure in a chamber of a Vigor MW oven (200 W, 30 min, 125 °C; Vigor, Moscow, Russia).

#### 4.1.2. HKUST-1*solv.1*

A solvothermal HKUST-1 sample (denoted as HKUST-1*solv.1*) was prepared by a modification of a reported method [66]. In a typical synthesis, Cu(NO_3_)_2_ 3H_2_O (8.58 mmol) was dissolved in H_2_O (20 mL), while H_2_BTC (0.525 g) was dissolved in absolute ethanol (15 mL). Then, both solutions were transferred into a Teflon autoclave and heated in an oven at 125 °C for 24 h. The product was filtered under a vacuum and dried at 155 °C overnight.

#### 4.1.3. HKUST-1*solv.2*

A solvothermal HKUST-1 sample (denoted as HKUST-1*solv.2*) was prepared according to the original procedure. In a typical synthesis Cu(NO_3_)_2_ 3H_2_O (8.58 mmol) was dissolved in H_2_O (20 mL), while H_2_BTC (0.525 g) was dissolved in absolute DMF (15 mL). Then, both solutions were combined and heated in an oil bath at 104 °C for 30 min. The product was filtered under a vacuum and dried at 155 °C overnight.

### 4.2. Characterization of HKUST-1 Materials

#### 4.2.1. N_2_ Adsorption Data

These data were obtained at 77 K using an 02 ASAP 2020 Plus instrument (Micromeritics, Norcross, GA, USA). The specific surface areas were calculated according to the BET equation. The total pore volume was evaluated at *p*/*p*_0_ = 0.99. The distribution in sizes of mesopores was calculated from the desorption branch according to the method of Barrett, Joyner and Halenda (BJH). The cumulative volume at desorption in the BJH method was taken as the mesopore volume. The adsorption film on the mesopore surface was taken into account. The micropore volume was calculated as the difference between the total pore volume and the mesopore volume. The micropore size distribution was calculated according to the Horwath–Kawazoe model in assumption of a cylinder shape of the pores [67].

In order to probe the mechanical robustness of the HKUST-1*mw* material, a portion of this sample synthesized under MW-irradiation and activated as it was described above was transferred in the molding form and pressurized under 20 atm (air) using a hydraulic pellet press (Perkin-Elmer, New Brunswick, NG, USA) maintaining this pressure during 2 min. The pressurized HKUST-1*mw* press sample was divided into the granules, and the N_2_-low temperature adsorption was measured for them.

#### 4.2.2. SEM Analysis

Sample morphology was studied using a SU8000 field-emission scanning electron microscope (FE-SEM, Hitachi, Mannheim, Germany). The target-oriented approach was utilized for the optimization of the analytical measurements [68]. The samples were mounted on a 3 mm copper grid with a carbon film and fixed in a grid holder. Images were acquired in the bright-field STEM mode at a 30 kV accelerating voltage.

#### 4.2.3. Powder X-ray Diffraction

X-ray powder diffraction data were collected in a reflection mode using an EMPYREAN instrument (PANalytical, Malvern, United Kingdom) equipped with a linear X’celerator detector and non-monochromated Cu Kα radiation (α = 1.5418 Å), measurement parameters: tube voltage/current 40 kV/35 mA, divergence slits of 1/16 and 1/8°, 2θ range 5–50°, speed 0.1° min^−1^.

### 4.3. Measurements of the Adsorption Selectivity in the HPLC Tests

The elution of the extremely dilute solutions of the studied compounds was carried out in the isocratic mode with pure methanol and acetonitrile at a volumetric flow rate of the mobile phase *F* = 500 μL/min and temperature 35 °C using a Agilent 1200 Series liquid chromatograph (Agilent Technologies, Waldbronn, Germany) equipped with a diode array detector, a Rheodyne dispensing valve (Waters, Milford, MA, USA) with a loop (20 μL) and thermostated columns. HKUST-1 powder as a suspension in chloroform was packed in the steel chromatographic columns by a pneumatic pump (Alltech, Weingarten, Germany). This pump was connected to a special cylindrical tank for a suspension with a volume of 25 mL. When packing the column, using a manometer, we ensured that the working pressure in the system did not exceed 200 atm. Before filling into the column, a suspension of HKUST-1 powder in chloroform was decanted. Moreover, we filtered off coarse particles on a copper mesh and treated this suspension with an ultrasonic rod (1–2 min). Chloroform was used as a solvent when filling a suspension into the column. The dimensions of the column filled with HKUST-1 granules were 50.0 × 4.6 mm. A free (dead) column volume *V*_M_ (μL) was determined by the minor disturbance method [60]. The adsorbates were detected at a wavelength of the detector λ = 254 nm. Examples of the output curves recorded during the elution of some chlorophenols at 65 °C through the porous HKUST-1 layer is presented in Figures S1–S4.

The adsorbate retention time *t*_R_ (min) was determined on a maximum of the peak of the output curve. Further, the corrected retention volume *V*_R_’ (μL) of the adsorbate was calculated by the formula: *V*_R_’ = *V*_R_ − *V*_M_ = *t*_R_·*F* − *V*_M_, where *V*_R_—retention volume of the adsorbate, μL. The shapes of the output curves measured in the HPLC experiments were close to the correct Gaussian peak shape. Therefore, it makes possible to calculate the Henry constant of the adsorption *K*_1,c_ (μL/m^2^) from the measured chromatographic values according to the known formula [62]: *K*_1,c_ = *V*_R_’/*S*_BET_·*g*, where *S*_BET_—the specific surface area (m^2^/g) of the HKUST-1 material, *g*—the mass of the adsorbent in the column (g). The Henry constant of the adsorption reflects the affinity between the adsorbate molecule and the adsorbent surface. Henry constants of the adsorption *K*_1,c_ of the studied compounds (*T* = 308 K) calculated by this formula are presented in Table 1.

### 4.4. Study of the Mechanism of Liquid-Phase Adsorption onto HKUST-1 Samples

The impact of the mobile phase composition on the benzene retention on the HKUST-1 adsorbents was studied as follows. Before HPLC measurements, the columns packed with HKUST-1 particles were rinsed with a mobile phase of different composition at 35 °C and eluent flow rate 500 µL/min for 40 min. We used the extremely dilute benzene solutions in the examinations of the liquid-phase adsorption mechanism. Thus, the liquid-phase adsorption was studied under conditions of extremely low degrees of the coverage of the adsorbent surface (the so-called Henry range). The retention times for benzene were obtained under these constant conditions and at the following volumetric ratios of *n*-hexane to isopropanol in the eluent: 99.9/0.1, 99.5/0.5, 99/1, and 95/5% (vol.). The volume of the probe injected in the chromatographic system was 20 µL.

In order to study adsorption processes in HPLC occurring in the HKUST-1 pores, the dependencies of the benzene retention on the flow rate of the mobile phase were obtained. The tests were carried out at the constant eluent composition (99.5% *n*-hexane and 0.5% isopropanol) and temperature 35 °C. The retention times of benzene are measured at different times of eluent flow rates: 50, 100, 300, 400, and 500 µL/min.

For the determination of thermodynamic characteristics of benzene adsorption onto HKUST-1 samples, we measured the dependencies of the benzene retention times during HPLC adsorption on the column temperature. The column temperature was changed in the range of 35–65 °C with a step of 10 °C under other equal conditions. The flow rate of the mobile phase was 50 µL/min. The thermodynamics of benzene adsorption was studied from pure *n*-hexane as well as a *n*-hexane–*iso*-propanol binary mixture with the *n*-hexane to isopropanol ratio of 99.5/0.5%. When changing the temperature before the probe injection, each column was thermostated for 20 min for achievement of the equilibrium.

The linear dimensions of both columns filled with HKUST-1 microparticles were 50.0 × 4.6 mm. A free (dead) column volume *V*_M_ (μL) was determined by the minor disturbance method and direct weight method [60]. Using the free column volume values obtained by the minor disturbance method (because they are smaller than the values determined by the direct weight method), we have calculated the retention factor (*k*), distribution constant (*K*_c_), enthalpy (∆*H*°), and entropy (∆*S*°) of the adsorption by the following well-known formulae (1)–(3) [62]:

*k* = (*V*_R_ − *V*_M_)/*V*_M_ (1)

*K*_c_ = *kV*_M_/*V*_S_ + 1 (2)

ln*K*_c_ = −∆*H*°/*RT* + ∆*S*°/*R* (3)

where *V*_S_ is the volume of the adsorbent in the chromatographic column, μL; *R* is the universal gas constant, J/(mol K); *T* is the absolute thermodynamic temperature, *K*.

The values of the standard enthalpy and entropy of the liquid-phase adsorption calculated by this method reflect changes in the energy and number of degrees of the freedom of the thermodynamic system during adsorption.

## 5. Conclusions

The selectivity of the liquid-phase adsorption of the structurally related compounds including structural isomers of aromatic compounds on the microporous HKUST-1 framework was studied by the HPLC technique. In order to study an effect of the preparation procedure on the physico-chemical characteristics of the HKUST-1 samples, they were synthesized by the original MW-assisted procedure and a “classical” solvothermal technique. An influence of the MW-irradiation on the structural characteristics of the HKUST-1 material was demonstrated by the XRD method.

It has been shown that the adsorption selectivity for the HKUST-1 material under liquid-phase conditions is determined both by the size and shape of the adsorbate. These geometrical parameters contribute to the penetration of the adsorbate molecules in the pores of the HKUST-1 matrix as well as their binding strength with the pore surface. The functional groups, such as nitro- and N-heterocyclic moieties in the adsorbed molecules assist also to their interaction with adsorption sites of the HKUST-1 adsorbent. The results obtained show that using HKUST-1 material as a selective adsorbent allows one to reach a maximal fitting of the size and shape of the adsorbate to the geometrical parameters of the pores, which determines the strength of adsorbed compounds binding. Additionally, the nature of the liquid medium contributes also to the adsorption properties of this metal-organic matrix. This work results allow one to predict and optimize the separation of the multicomponent mixtures of organic compounds on the HKUST-1 matrix by the HPLC technique.

## Figures and Tables

**Table 1:** Henry constants of adsorption of organic compounds from their solutions in methanol and acetonitrile on the HKUST-1 metal-organic framework (HKUST-1*mw* sample).

Entry	Adsorbate	Structural Formula	Henry Constants of Adsorption *K*_1,c_, μL/m^2^	
			MeOH	MeCN
1	4-Chlorophenol	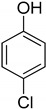	0.286	2.990
2	4-Chloro-2-methylphenol	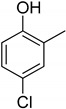	0.234	0.343
3	3,5-Dimethylphenol	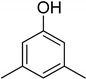	0.055	0.394
4	2,4-Dichloro-3,5-dimethylphenol	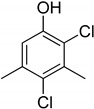	0.215	0.335
5	2,3-Dichlorophenol	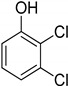	0.087	0.766
6	2,4-Dichlorophenol	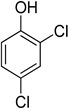	0.249	0.954
7	2,5-Dichlorophenol	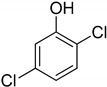	0.263	0.201
8	2,6-Dichlorophenol	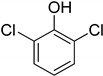	0.123	0.517
9	3,4-Dichlorophenol	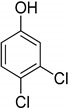	0.266	0.962
10	3,5-Dichlorophenol	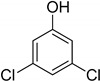	0.217	0.505
11	2,3,5-Trichlorophenol	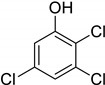	0.201	0.763
12	2,3,6-Trichlorophenol	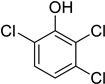	0.236	0.398
13	2,4,5-Trichlorophenol	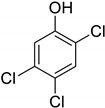	0.240	0.432
14	2,4,6-Trichlorophenol	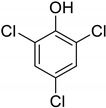	0.186	0.425
15	3-Nitrophenol	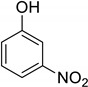	0.190	1.012
16	4-Nitrophenol	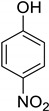	0.245	3.210
17	2,4-Dibromophenol	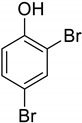	0.098	0.914
18	Tetracycline	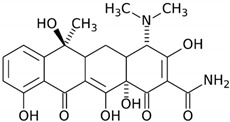	0.518	0.271
19	Tropic acid	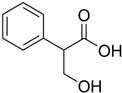	0.120	0.287
20	Ibuprofen	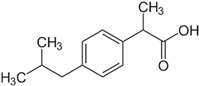	0.078	0.236
21	Paracetamol	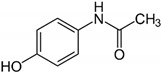	0.081	1.643
22	Diphenylamine	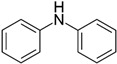	0.086	7.408
23	Nicotin	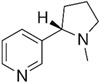	2.583	0.183
24	Benzotriazole	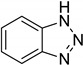	1.137	0.285

**Table 2:** Textural characteristics of the HKUST-1*mw*, HKUST-1*mw* press and HKUST-1*solv.1* samples.

Material	S_BET_ m^2^/g	V_total,_^a^ cm^3^/g	V_micro,_^b^ cm^3^/g	V_meso,_^c^ cm^3^/g	Pore Width, nm
HKUST-1*mw*	1617	0.767	0.679	0.088	0.4–0.8
HKUST-1*mw* 20 atm press	1613	0.778	0.695	0.083	0.4–0.8
HKUST-1*solv.1*	1648	0.816	0.714	0.102	0.6–0.9

^a^ V_total_ was estimated from the adsorption value at *p/p*_0_ = 0.99; ^b^ *V_μ_* = *V_Σ_* =*V_meso_*; ^c^ The cumulative mesopore volume was calculated from the desorption branch of the isotherm by the BJH method, and the standard thickness of the adsorption film.

## Supplementary Materials

The following are available online at https://www.mdpi.com/1420-3049/25/11/2648/s1, Figures S1–S4: Output curves recorded during the elution of some chlorophenols at 65 °C through the porous HKUST-1 layer.
