# Supplementary material for: Impact of the Preparation Procedure on the Performance of the Microporous HKUST-1 Metal-Organic Framework in the Liquid-Phase Separation of Aromatic Compounds
Source: Molecules. 2020 Jun 6;25(11):2648. doi: 10.3390/molecules25112648 (PMC7321157; doi:10.3390/molecules25112648)
Supplement: Supplementary file 1 [file molecules-25-02648-s001.pdf]

Supplementary Materials

Impact of the Preparation Procedure on the Performance of the Microporous HKUST-1 Metal-Organic Framework in the Liquid-Phase Separation of Aromatic Compounds

Vera I. Isaeva [1,2,*], Bulat R. Saifutdinov [3,*], Vladimir V. Chernyshev [3,4], Vadim V. Vergun [1], Gennady I. Kapustin [1,2], Yulia P. Kurnysheva [3], Mikhail M. Ilyin [5] and Leonid M. Kustov [1,2]

1 N.D. Zelinsky Institute of Organic Chemistry, Russian Academy of Sciences, Leninsky prospect 47, Moscow 119991, Russia; polubrat@mail.ru (V.V.V); gik@ioc.ac.ru (G.I.K.); lmkustov@mail.ru (L.M.K.)
2 National University of Science and Technology MISiS, Leninsky prospect 4, Moscow 119991, Russia
3 A.N. Frumkin Institute of Physical Chemistry and Electrochemistry, Russian Academy of Sciences, Building 4, 31 Leninsky prospect, Moscow 119071, Russia; vladimir@struct.chem.msu.ru (V.V.C.); kurnyshevay@mail.ru (Y.P.K.)
4 Department of Chemistry, M.V. Lomonosov Moscow State University, 1-3 Leninskie Gory, Moscow 119991, Russia
5 A.N. Nesmeyanov Institute of Organoelement Compounds, Russian Academy of Sciences, 28 Vavilov St., Moscow 119991, Russia; mil@ineos.ac.ru (M.M.I.)
* Correspondence: sharf@ioc.ac.ru (V.I.I.); bulatrsaifutdinov@gmail.com (B.R.S.)

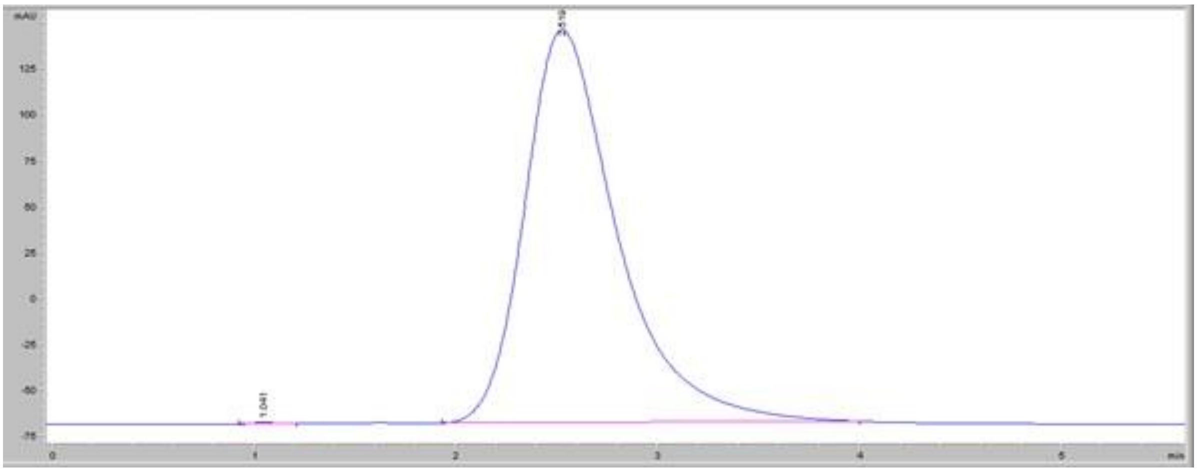

Figure S1. Output curve recorded during the elution of 2,3-dichlorophenol at 65°C through the porous HKUST-1 layer.

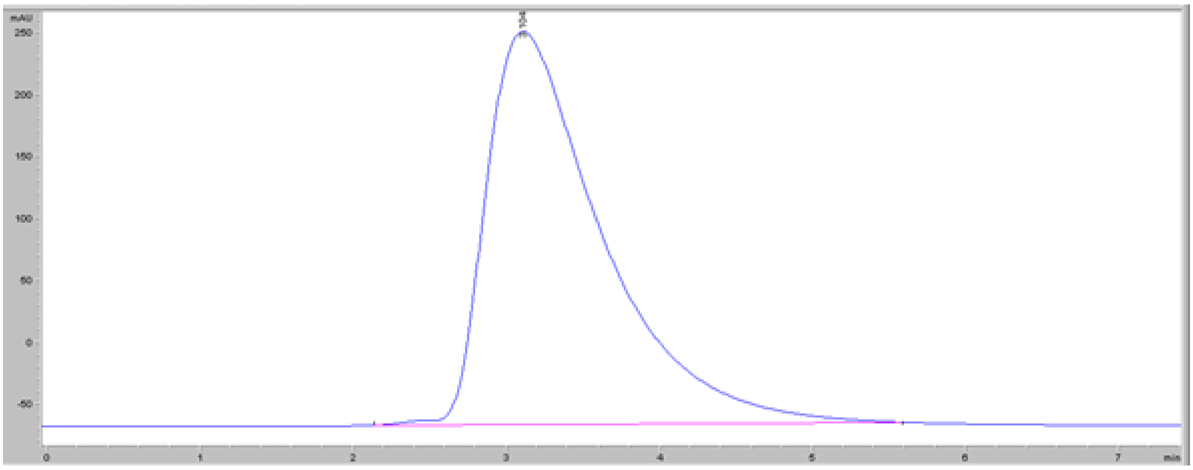

Figure S2. Output curve recorded during the elution of 2,4-dichlorophenol at 65°C through the porous HKUST-1 layer.

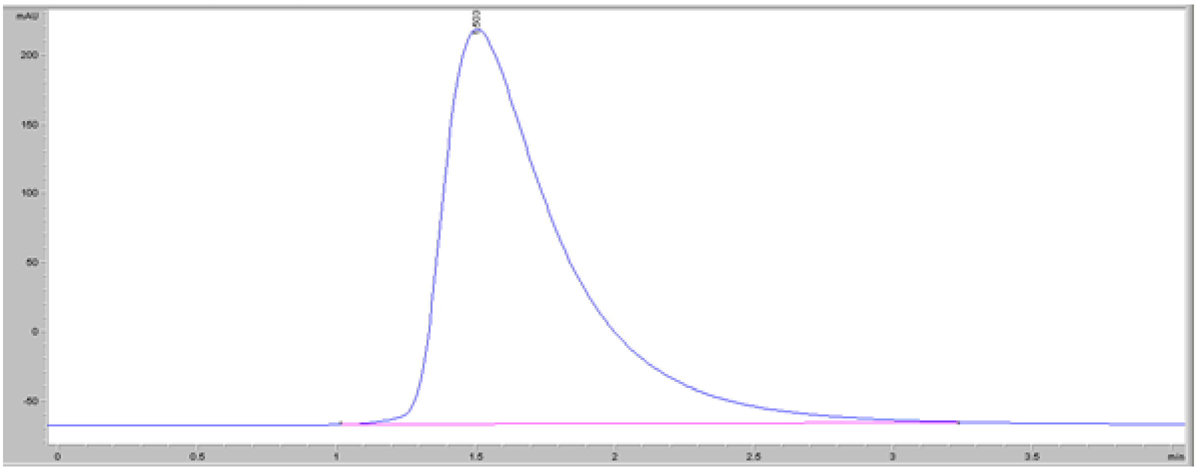

Figure S3. Output curve recorded during the elution of 2,5-dichlorophenol at 65°C through the porous HKUST-1 layer.

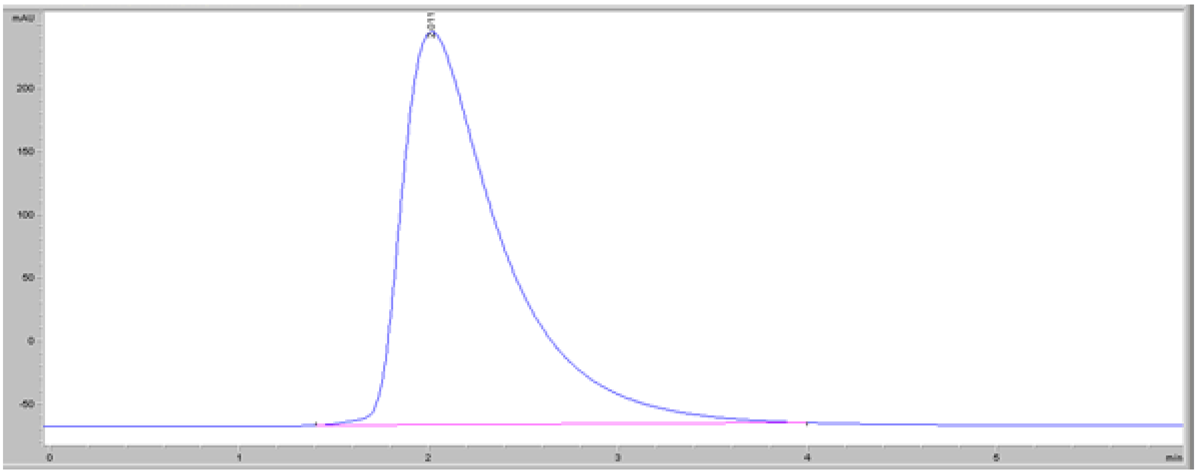

Figure S4. Output curve recorded during the elution of 2,6-dichlorophenol at 65°C through the porous HKUST-1 layer.
